# Soft Tissue Fibromyxoma of Non-odontogenic Origin and Its Management With Dental Lasers: A Case Report

**DOI:** 10.7759/cureus.66975

**Published:** 2024-08-16

**Authors:** Kowsalya Nallathambi, Ramnath Elangovan, Selvakumar J, Ebenezer Jayden, Dinesh C Maganti, Dona Soman

**Affiliations:** 1 Department of Periodontics, Adhiparasakthi Dental College and Hospital, Melmaruvathur, IND; 2 Department of Periodontology, School of Dentistry, University of Rwanda, Kigali, RWA; 3 Department of Periodontology and Community Dentistry, School of Dentistry, University of Rwanda, Kigali, RWA; 4 Department of Prosthetic and Restorative Dentistry, School of Dentistry, University of Rwanda, Kigali, RWA

**Keywords:** differential diagnosis, oral fibromyxoma, myxoma, soft tissue, benign tumors, intraoral myxoma

## Abstract

Myxomas are a group of benign tumors that have a common histologic appearance of fibrous and myxoid ground substance. According to literature, myxomas often occur between the ages of 30 and 50 years. Very often, intraoral soft tissue myxoma can be misinterpreted as malignant and is difficult to differentiate from other tumors with myxoid stroma. Of the different variants of soft tissue myxoma, intraoral is an extremely rare, slow-growing, benign ectomesenchymal tumor. We report a case of a 75-year-old female who presented with soft tissue swelling in the upper front tooth region. No history of pain over the lesion or bleeding was seen during brushing. On intraoral examination, a lesion measuring 2 x 3 cm was seen in the interdental papilla involving the attached gingiva of 22 and 23. An excisional biopsy of the lesion using a diode laser followed by low-level laser therapy revealed oral soft tissue fibromyxoma without odontogenic origin. A case of oral soft tissue myxoma is presented for its rarity and differential diagnosis of localized oral cavity lesions.

## Introduction

Myxomas are benign, uncommon tumors that can occur in various tissues, such as the bone, heart muscle, skin, subcutaneous and aponeurotic tissues, oral cavity, and urogenital tract. Among other intraoral and perioral connective tissue tumors, soft tissue myxomas are very rare and less frequent than odontogenic myxomas of the jaws. The term "myxoma" was originally used by Virchow in 1863 to describe mucinous tumors derived from the umbilical cord [1]. The initial case of a true jaw-odontogenic myxoma was reported by Thoma and Goldman in 1947 [2]. Odontogenic myxomas usually manifest between the ages of 22.7 and 36.9 years old [1]. Odontogenic fibromyxoma is a rare, locally aggressive, slowly growing benign neoplasm that typically manifests between the second and third decades of life.

Fibromyxoma is an aggressively benign tumor with all or part of its stroma made up of loosely clustered fusiform cells, stellate cells, or spindle-shaped cell forms [3,4]. There are two forms of odontogenic fibromyxoma identified according to their location within the oral cavity: central fibromyxoma, which occurs mostly at the center bone, and peripheral fibromyxoma, which frequently occurs at the alveolar process maxilla. These are ectomesenchymal origin tumors with or without odontogenic epithelium involvement, according to the World Health Organization (WHO) classification [5].

Those that develop centrally inside the bone are more common as compared to others. They grow slowly but may invade surrounding bone and soft tissues, requiring extensive surgical intervention for their eradication. Tumor recurrence potential, together with its invasive nature, highlights that care should be taken during surgery.

On the contrary, peripheral odontogenic fibromyxomas develop in soft tissues, mainly at the alveolar process maxilla. Though less common, these tumors are usually not as aggressive as the central ones. They may present as soft, painless swellings that sometimes mimic other benign conditions, thereby resulting in delays in diagnosis and treatment. These peripheral myxomas are histologically close to other various myxoid tumors, but they are benign in nature. Hence, an accurate histopathological diagnosis is essential for proper management of the condition.

## Case presentation

A 75-year-old female reported to the Department of Periodontics and Implantology, Adhiparasakthi Dental College and Hospital, Melmaruvathur, with a chief complaint of growing gums in the upper left front tooth. The patient was apparently normal and noticed a tissue mass that had developed a few months ago, involving the interdental papilla between the maxillary left lateral incisor and the canine (22 and 23). The swelling was painless, and on inquiry, the patient reported no history of trauma at the site. The swelling was initially small but gradually increased in size over the past year.

**Figure 1:**
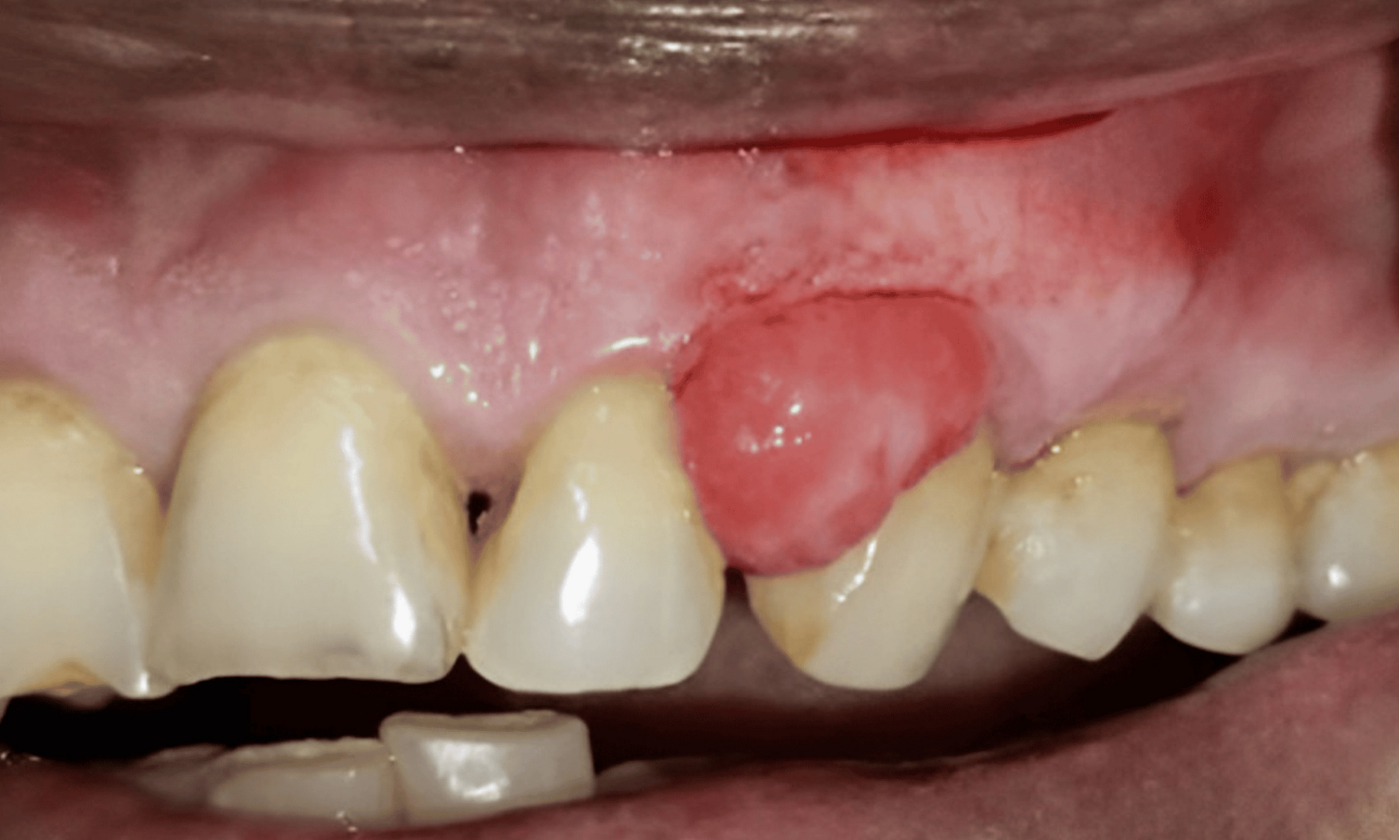
Observation of the lesion in the gingiva before to the operation

The patient recorded that there was occasional bleeding during brushing from the soft tissue mass. Medical history revealed the patient has been on medication for hypertension for the past 15 years (amlodipine 5 mg). There were no significant findings on the extraoral examination. Regional lymph nodes were not palpable. Intraoral examination showed a solitary, reddish, painless, sessile nodule of soft consistency that measured approximately 2 x 3 cm (Figure 1).

**Figure 2:**
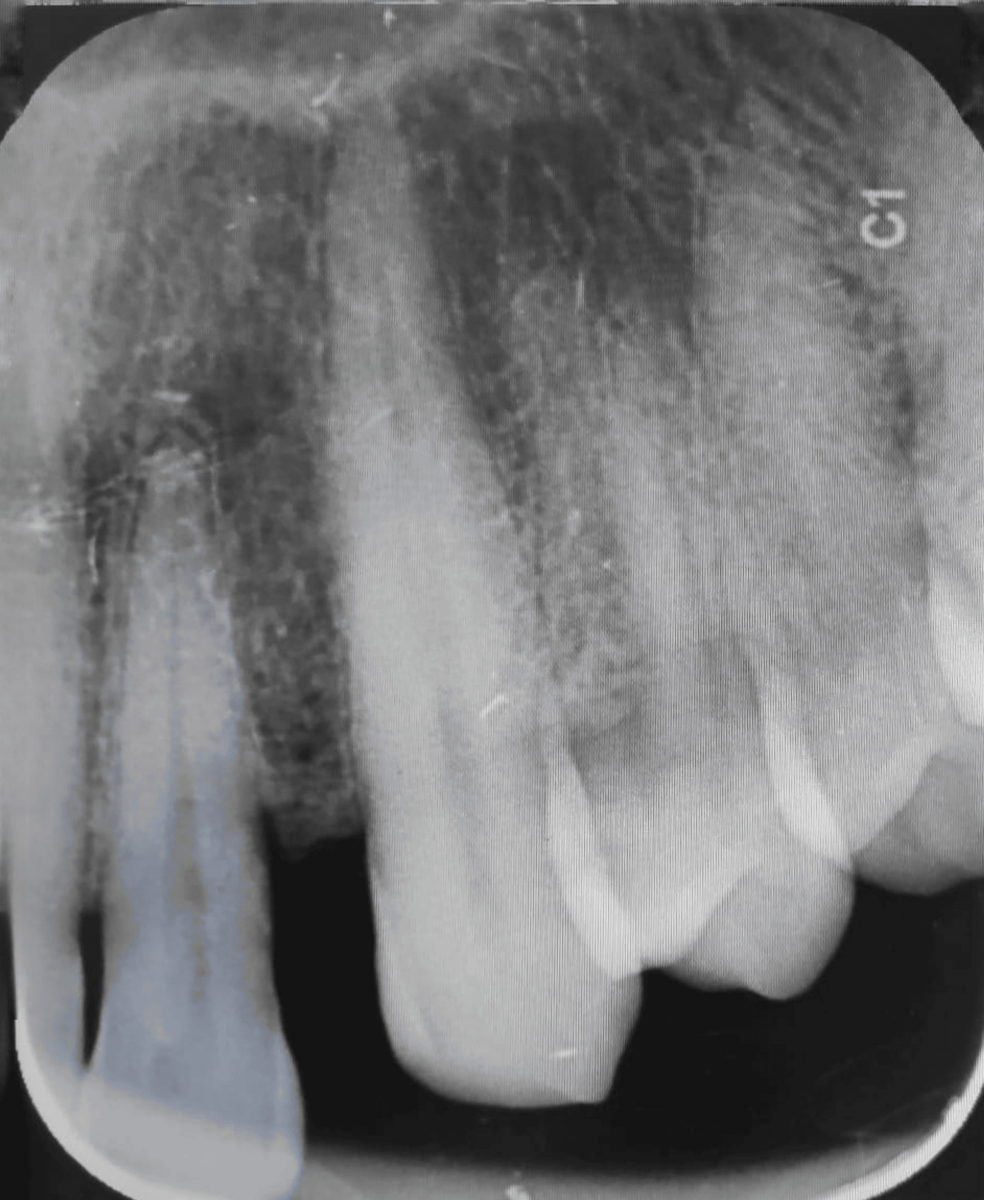
An intraoral periapical radiograph of the area affected by the lesion

The involved tooth was found to be periodontally sound and noncarious. Clinically, the tooth was firm. Oral hygiene maintenance was inadequate, and generalized bleeding on probing was present. Radiographic examination revealed an open contact area between 22 and 23, no periapical pathology was present, and the tooth was vital (Figure 2).

**Figure 3:**
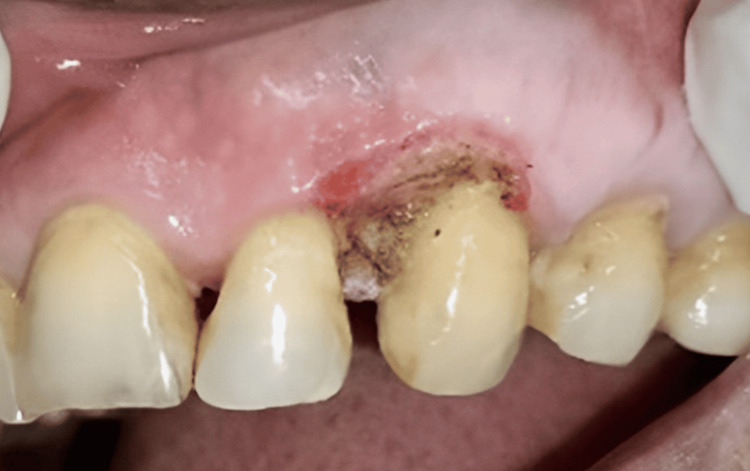
The removal of the lesion using surgical means by the use of lasers (immediately after excision)

A provisional diagnosis of pyogenic granuloma was made and the patient was subjected to phase I therapy, which included patient motivation, patient education, and oral prophylaxis. Following phase 1 therapy, surgical excision of the lesion was planned. Under adequate local anesthesia (2% lignocaine, 1:1,00,000 epinephrine). The lesion was excised with a diode laser (Indilase 980 nm, 1 w) in gated pulsed mode (Figure 3).

**Figure 4:**
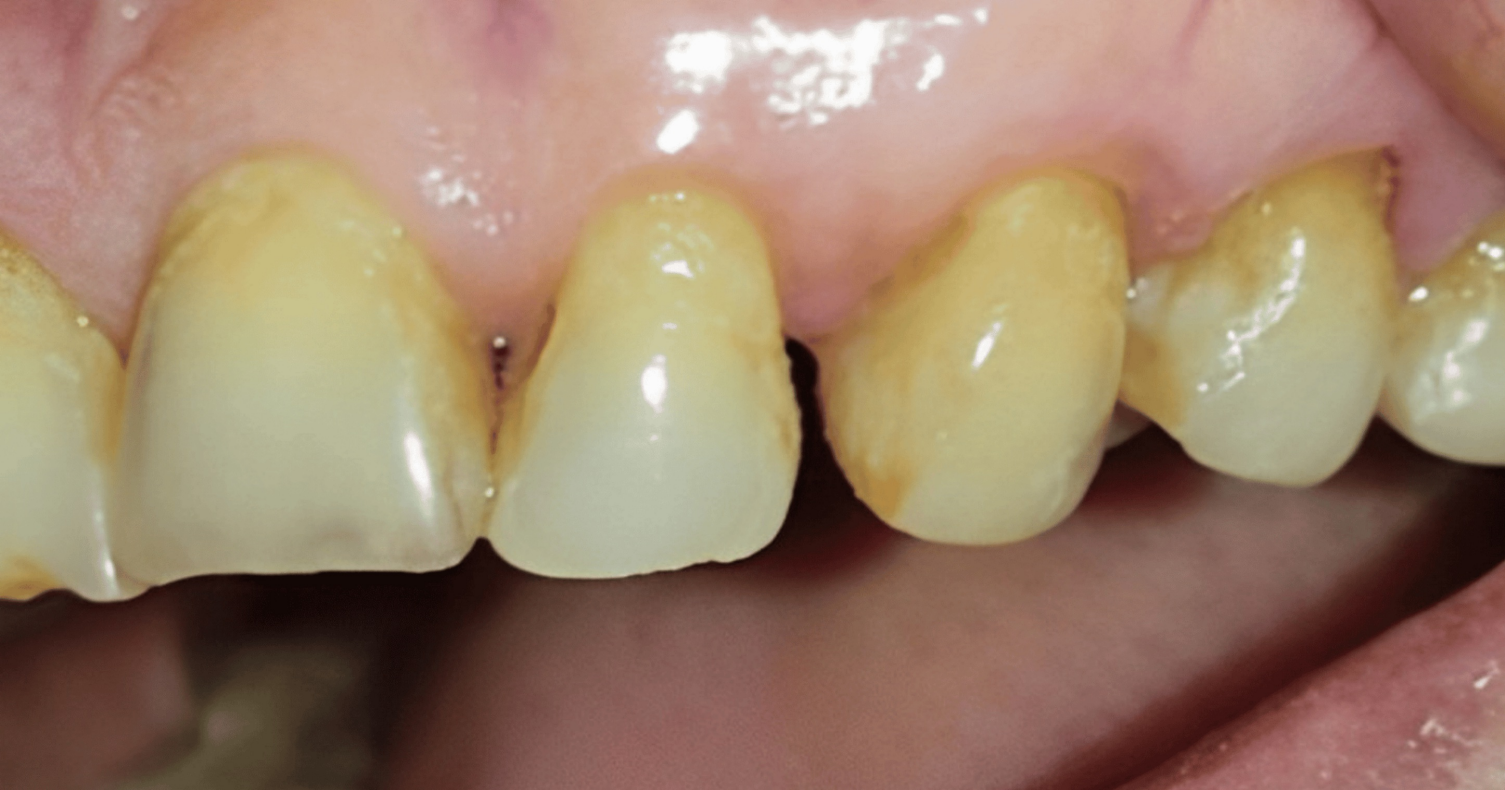
One week follow-up of the lesion after the surgery

**Figure 5:**
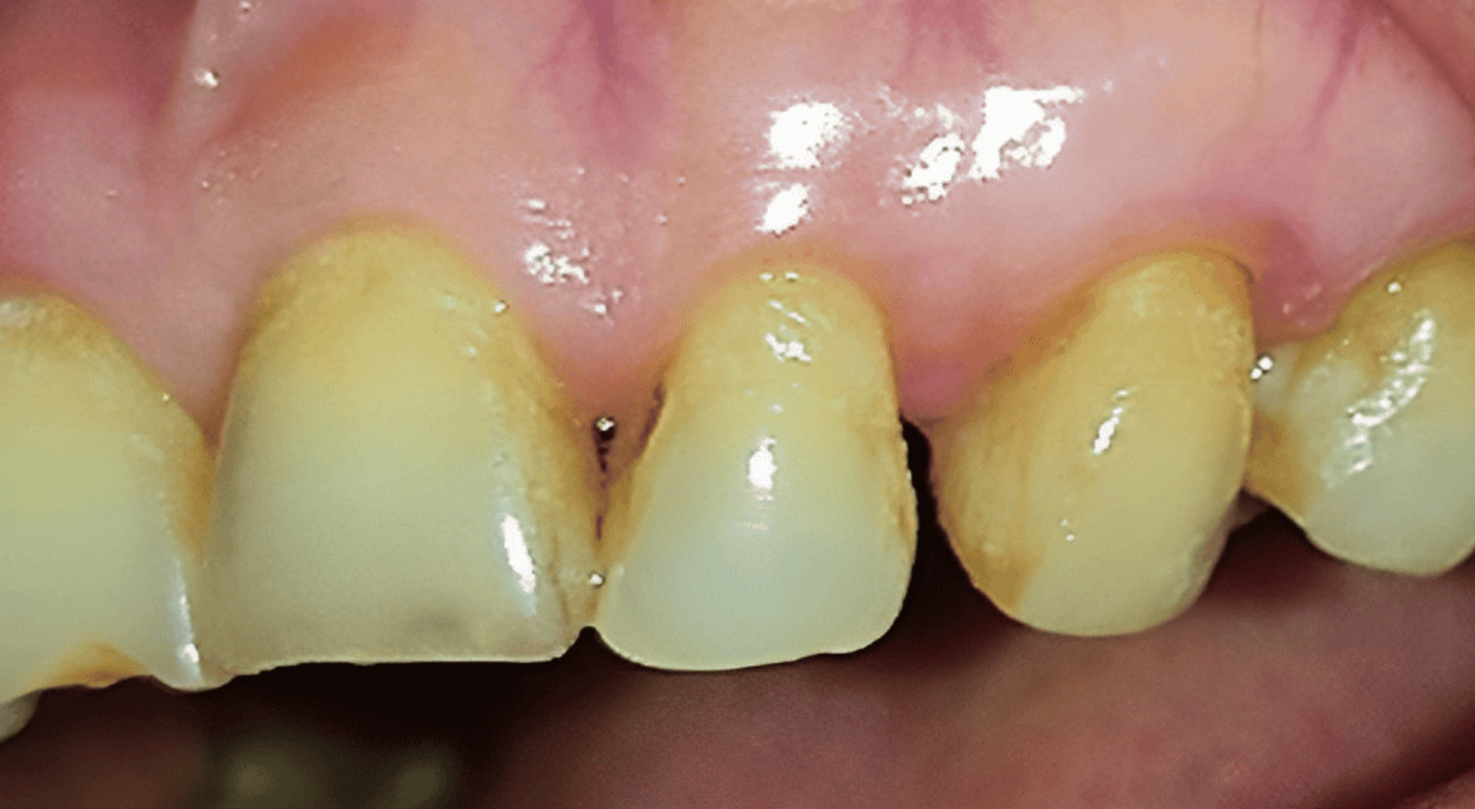
Six month follow-up of the lesion after excision

In order to prevent recurrence, and for histopathological purposes, a small amount of normal tissue around the lesion was excised. The excised tissue was transferred to a 10% formalin solution and sent for histological examination. Following the excision, there was no bleeding, and the excised area was subjected to low-level laser therapy (LLT) with a setting of 780 nm, 60 mW, and 3.0 J/cm2. After surgery, no antibiotics were administered, and the patient was advised to take analgesics (ibuprofen 400 mg) as required. The patient was recalled after a week (Figure 4) and after six months (Figure 5) for review.

**Figure 6:**
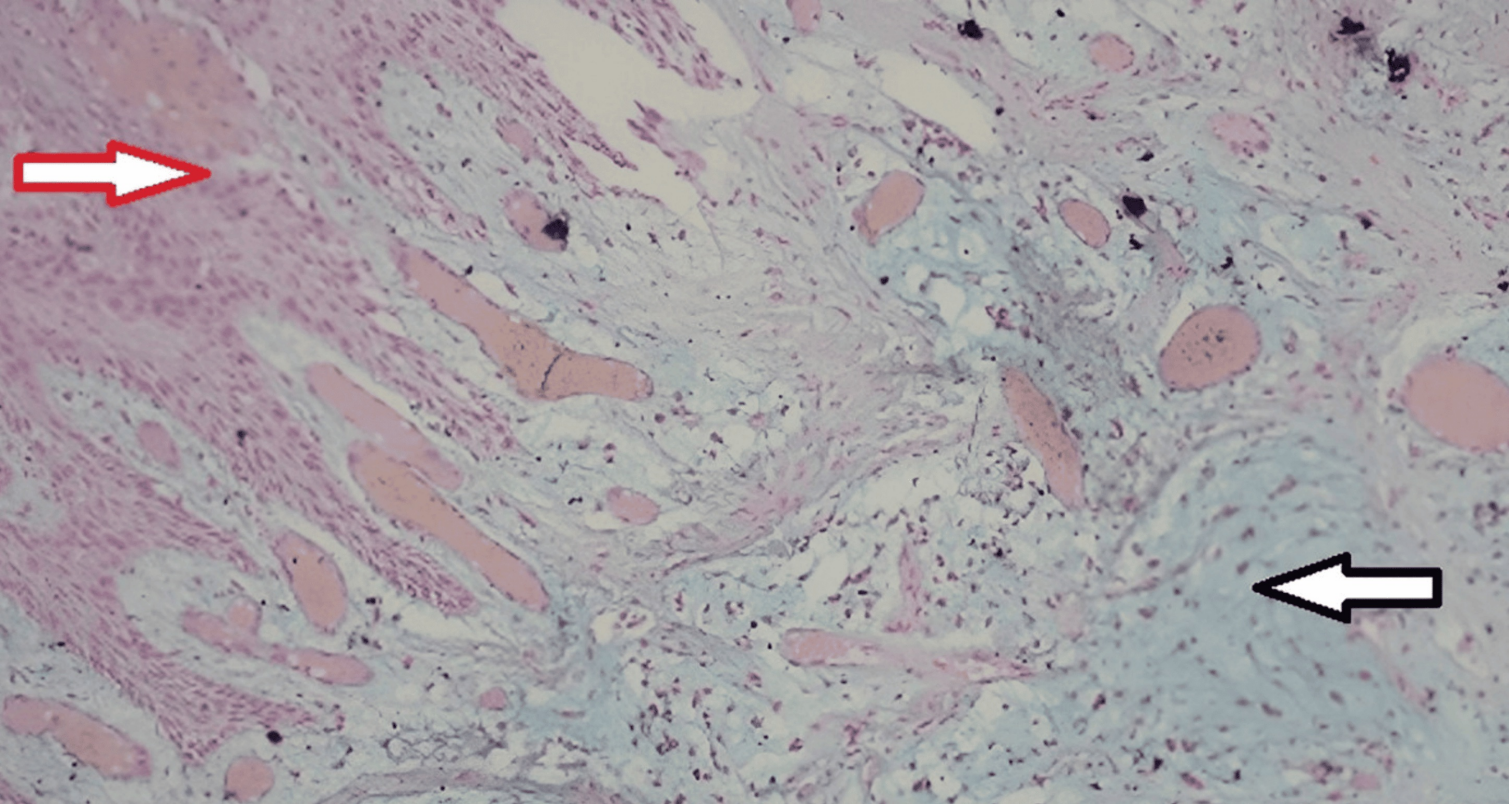
Masson's Trichrome-stained section under 10x magnification reveals connective tissue areas with fibrous component (red arrow) and myxomatous component (black arrow) with dispersed blood vessels and inflammatory cells

During these reviews, the healing was uneventful without any postoperative complications, and there was no recurrence at six months. However, the Masson's Trichrome-stained sections revealed findings of connective tissue areas with fibrous components and the myxoid component with dispersed blood vessels and inflammatory cells, leading to a provisional diagnosis of pyogenic granuloma (Figure 6).

**Figure 7:**
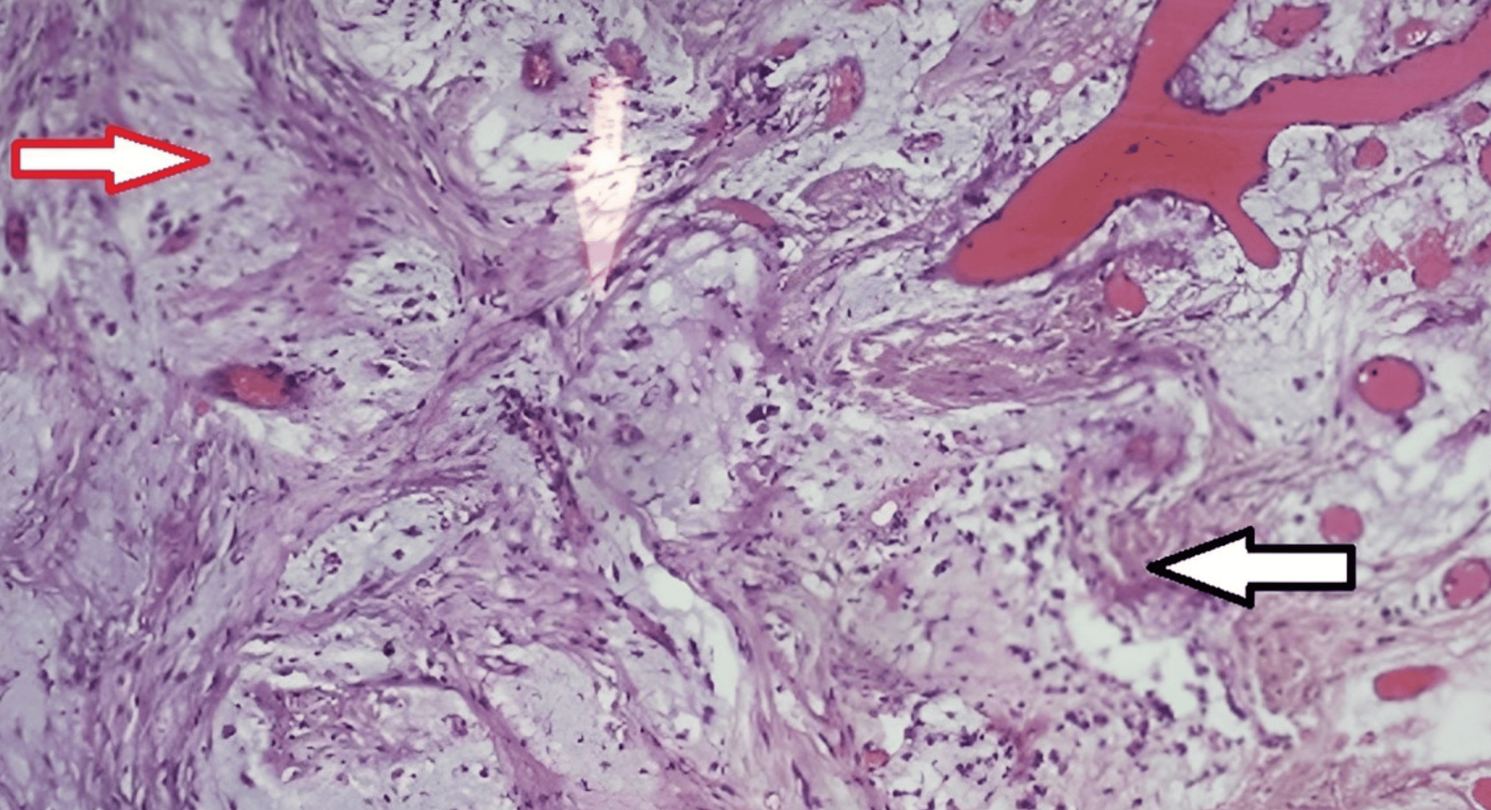
Special stain: Alcian blue-stained section under 10x magnification reveals connective tissue areas with fibrous component (black arrow) and myxomatous component which is bluish green stained (red arrow) with dispersed blood vessels and inflammatory cells. Overlying hyperplastic stratified squamous epithelium is seen

Since myxomas arise from ectomesenchymal origin and have abundant myxoid tissue made of mucins and glycosaminoglycans in the ground substances, special staining using Alcian blue was used. The Alcian blue staining demonstrated connective tissue areas with fibrous and myxoid components containing numerous inflammatory cells and dispersed blood vessels (Figure 7). Based on the histopathological findings and clinical correlations, the final diagnosis of soft tissue fibromyxoma without odontogenic origin was made.

## Discussion

Myxomas make up between 2.3% and 17.7% of all odontogenic tumors, with fibromyxomas being an insignificant component of myxomas. Odontogenic peripheral myxofibroma is a benign neoplasm without odontogenic epithelial involvement that is locally aggressive and frequently recurs in the oral cavity. Few cases of odontogenic fibromyxoma have been reported in the literature [6-9]. The method to treat this condition is excision, depending on the extent and involvement of the bone. The lesion presents in a variety of ways. Firstly, several reports on gingiva have shown no association with odontogenesis, despite the presence of soft tissue myxoma [10,11].

Several reports suggest that females are more susceptible to developing soft tissue fibromyxomas at a ratio of 2.5:1 relative to males, with the mandible being more affected by this than the maxilla [12]. In our case, it was located at the interdental papilla, involving the attached gingival region of teeth 22-23. A female patient aged 75 years had a history of hypertension and had been taking medication (amlodipine for the last 15 years. The initial diagnosis was drug-induced gingival enlargement due to the long-term use of amlodipine, known to cause gingival overgrowth as a calcium channel blocker. However, this possibility was eliminated by localization and normal appearance at other sites on the gum.

The most common lesion seen in this age group is pyogenic granuloma; hence, surgical excision followed by hematoxylin and eosin (H&E) staining was planned. Laser ablation combined with photobiomodulation was selected given the patient's elderly hypertensive status. A solution containing lignocaine (2%) mixed with epinephrine (1:100000) was preferred because it was thought to be safer for a patient with cardiovascular disease [13]. Compared to laser ablation combined with photobiomodulation, this solution led to improved healing outcomes and reduced postoperative pain for the patient. A check-up at six months did not show any recurrence of the lesion.

While odontogenic fibromyxomas are rare, they pose a significant clinical challenge due to their locally aggressive behavior and high rates of recurrence. These tumors, although benign, have been known to exhibit behavior that calls for extensive surgical management. Enucleation is often preferred as the best treatment, particularly when there is wide bone involvement. The scarcity of peripheral soft tissue myxomas not involving the odontogenic epithelium contributes another level of complexity to diagnosis and management. It is important to consider broad differential diagnoses when evaluating gingival lesions, even in elderly complex medical history patients. Initially, drug-induced gingival enlargement was suspected due to the long-term use of amlodipine, which is known for causing gingival overgrowth since it is a calcium channel blocker. However, this hypothesis was ruled out because only one gum had an abnormality without any other signs on the rest of the gum.

The choice of laser excision for this type illustrates benefits derived from using less invasive surgical techniques, especially in very old people with pre-existing diseases. Some key benefits attached to laser surgery include reduced intraoperative bleeding, minimized postoperative discomforts, and improved healing processes. Photobiomodulation also played a role through low-intensity lasers, stimulating cellular activity and promoting tissue repair, leading to the improved outcome observed in this patient. The positive outcome after six months of follow-up without recurrence demonstrates that the selected treatment approach works. It further highlights that individualized care, while considering overall health status, age, and presentation, matters most during the process of handling such cases.

The case report adds to the small number of publications on peripheral soft tissue fibromyxomas and stresses how critically doctors should treat such rare cases. It shows that laser excision and photobiomodulation can be used to treat benign but potentially aggressive cancerous tissues that are confined in the mouth, especially among patients with complicated medical histories. Therefore, more studies and case reports should be done so as to get a better understanding of their pathogenesis, diagnosis, and treatment, which will eventually help in enhancing patients’ outcomes as well as expanding our knowledge in this field.

## Conclusions

This case report discussed the rare presentation of soft tissue fibromyxoma without odontogenic origin in a patient with 15 years of hypertension. The management was done by using a diode laser. Therefore, the application of diode lasers in the excision of oral lesions is very efficient, as it is bloodless and has no postoperative complications. Photobiomodulation increases the healing of the tissues and reduces postoperative pain by masking the peripheral nerve endings. To conclude, proper diagnosis and early treatment with the best available treatment options are necessary for the successful management of oral benign lesions.
